# Field Programmable Gate Array-Embedded Platform for Dynamic Muscle Fiber Conduction Velocity Monitoring

**DOI:** 10.3390/s19204594

**Published:** 2019-10-22

**Authors:** Daniela De Venuto, Giovanni Mezzina

**Affiliations:** Department of Electrical and Information Engineering, Politecnico di Bari, 70125 Bari, Italy; daniela.devenuto@poliba.it

**Keywords:** MFCV, EMG, FPGA, Real-time EMG detection

## Abstract

This paper proposes a novel architecture of a wearable Field Programmable Gate Array (FPGA)-based platform to dynamically monitor Muscle Fiber Conduction Velocity (MFCV). The system uses a set of wireless sensors for the detection of muscular activation: four surface electromyography electrodes (EMGs) and two footswitches. The beginning of movement (trigger) is set by sensors (footswitches) detecting the feet position. The MFCV value extraction exploits an iterative algorithm, which compares two 1-bit digitized EMG signals. The EMG electrode positioning is ensured by a dedicated procedure. The architecture is implemented on FPGA board (Altera Cyclone V), which manages an external Bluetooth module for data transmission. The time spent for data elaboration is 63.5 ms ± 0.25 ms, matching real-time requirements. The FPGA-based MFCV estimator has been validated during regular walking and in the fatigue monitoring context. Six healthy subjects contributed to experimental validation. In the gait analysis, the subjects showed MFCV evaluation of about 7.6 m/s ± 0.36 m/s, i.e., <0.1 m/s, a typical value for healthy subjects. Furthermore, in agreement with current research methods in the field, in a fatigue evaluation context, the extracted data showed an MFCV descending trend with the increment of the muscular effort time (Rested: MFCV = 8.51 m/s; Tired: 4.60 m/s).

## 1. Introduction

One of the most challenging goals in the healthcare field currently concerns the improvement of the efficiency of healthcare infrastructures and related biomedical systems [1].

The current procedures for patient monitoring, care, management, and supervision are often fully left to the manual execution of hospital staff and in most cases are limited to non-exhaustive ambulatory analyses. Such procedures represent a bottleneck in term of healthcare efficiency [1,2] and could lead to errors in medical practices [2].

In this context, there is a significant need to deliver quality care to patients, to introduce more comfortable outpatient and at-home clinical protocols, and to explore the possibility of remote and continuous monitoring of useful health parameters.

Advances in wireless sensors networks and the Internet of Things (IoT) technologies have prompted the development of smart systems to support and improve healthcare and biomedical-related processes [1,2]. Together with the development of high-performance wireless protocols, the reduction in size of portable and wearable electronics [3,4] has ensured a number of solutions that non-invasively acquire bio-signals (e.g., electroencephalography and electromyography) and analyze them in situ to identify, classify, and prevent several pathologies [5] and disorders [6]. 

The solutions which combine these technological advances to improve healthcare efficiency constitute a research field commonly known as smart healthcare (s-Health).

In the s-Health context, we propose a cyber-physical platform for the remote monitoring of the progress of diabetes [4], allowing in-home use of a typical clinical protocol. 

In this work, we refer to the diabetic peripheral neuropathy (DPN), a pathology associated with vascular injuries typical of diabetic subjects, which leads to changes in a parameter known as muscle fiber conduction velocity (MFCV) [5]. The MFCV has an important clinical role in the progress assessment of several pathologies [5,6,7,8] and in muscle fatigue monitoring [8,9]. 

It has been proven [10] that MFCV can be reliably assessed non-intrusively by using surface electromyography (EMG) [10]. Specifically, it can be extracted by placing two surface electrodes along the same muscle fiber and deriving the motor unit action potential propagation speed by a linear model. The considered approximation consists of the uniform rectilinear motion model:

(1) $$D_{2}=D_{1}+v\vartheta$$

where D_2_ is the position of the second electrode along the muscle fiber, D_1_ refers to the first one, ϑ identifies the measure of the propagation time, and v the MFCV to be estimated. In the following we refer as D = D_2_ − D_1_ as the inter-electrode distance. 

Despite its clinical applicability, there are currently no solutions which are able to evaluate in real time MFCV during a dynamic task (i.e., ordinary life movements such as walking) and fatigue [8,11,12,13]. In most cases, this kind of solution requires cumbersome structure such as ergometer cycles [12,13], limiting their use to ambulatory applications. Other diffused solutions force the subject to keep long isotonic contraction to reliably extract MFCV [8,12,13]. Nevertheless, the measurement protocol on which the systems are based limits the comfort and freedom of movement for the subject.

Aiming to bridge this gap, a Field Programmable Gate Array (FPGA)-based cyber-physical platform, for remote monitoring of MFCV during dynamic ordinary life exercises, is proposed. The conduction velocity evaluation will concern subjects involved in walking (on-line MFCV assessment) and squat exercises (fatigue monitoring), achievable even in a domestic environment. 

The platform acquires data from four wireless EMG electrodes (two for each leg), which triggers MFCV measurement (cyclical movements requirement [14]), by using two footswitches inside the plantar of shoes. Exploiting a single XNOR-based bit stream cross-correlation algorithm, the platform can realize a MFCV measure in ~370 ms, with a ϑ resolution of 0.5 ms@2 kHz providing only 4 kbps to the central elaboration logic. The paper is structured as follow. First, Section 2 investigates the current state of the field, then provides an architectural overview, detailing its working principles. The same section presents the FPGA implementation of the method. Section 3 shows the experimental results in terms of ordinary life applicability and FPGA performance. Finally, Section 4 concludes the paper with some remarks.

## 2. Materials and Methods

### 2.1. State of the Field

The field literature proposes several designs [11,12,13,14,15] for reliable MFCV assessment through dedicated electronics. Among the designs that acquire and analyze in situ EMG bio-signals, noteworthy is a microcontroller-based platform as described in [11]. In [11], the microcontroller (68HC16) extracts the MFCV by implementing a cross-correlation between two electro-stimulated EMG signals. The invasive nature of the system (electrical stimulation) and the impossibility of the solution to provide a real-time MFCV assessment (computational time: 670 ms to achieve a ϑ resolution ~2 ms) makes it not suitable in outpatient applications. The authors in [8] propose an Application Specific Integrated Circuit (ASIC) for MFCV-based fatigue evaluation. The authors asked subjects to maintain an arm muscle in static contraction. The design in [8] reaches high ϑ resolutions (~0.1 ms @10 kHz), requiring a continuous data flow (140 kbps @10 kHz) to the central computing unit. It lead to a high computational effort for the digital processor. In addition, the proposed ASIC does not allow the muscle stress evaluation in dynamic exercises, forcing the subject to keep the contraction all along the measurements. 

All the above-described portable solutions concerned the MFCV assessment in electro-stimulated or static contraction exercises. The literature on MFCV estimation during dynamic exercises does not present, to the best of our knowledge, easily wearable and remotely monitoring solutions [12,13]. As previously stated, in all the proposed designs for dynamic evaluation, the limb movements are properly synchronized with the MFCV assessment system by using cumbersome machines (e.g., an ergometer cycle or fitness bench) [12,13]. It excludes the possibility of MFCV evaluation during the context of daily life. 

### 2.2. System Overview

**Figure 1:**
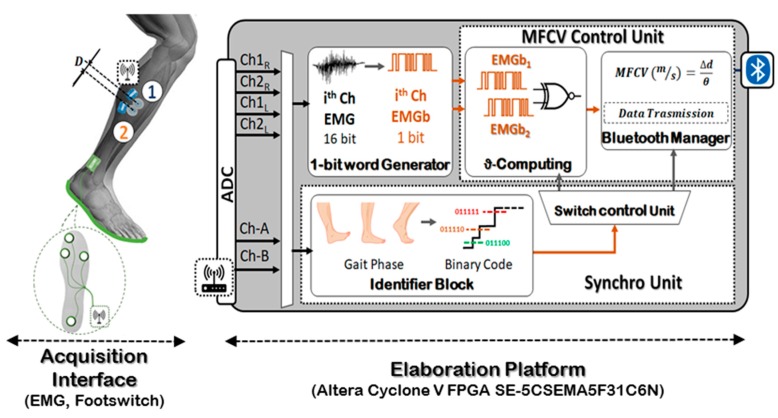
System architecture.

Figure 1 shows a top-level block diagram of the realized system. Following the workflow in Figure 1, the architecture can be divided into two main sub-systems: the Acquisition Interface and the fully FPGA-based Elaboration Platform. 

The Acquisition Interface consists of the bio-signals front-ends including four wireless sEMG (placed on lower limbs) and two wireless footswitches. This sub-system allows for the capture of signals associated to the muscle activity (via sEMG) and feet position information (via footswitches). The sensing nodes transfer the data to a gateway [15,16], which is directly connected to an FPGA evaluation board (Altera Cyclone V FPGA on DE1-SoC) that embeds the computation unit (Elaboration Platform). This latter block is involved in the MFCV evaluation when specific feet positions are detected. 

In the first step, the EMG data from the gateway are computed by a dedicated block named 1-bit word Generator, as seen in Figure 1. This block has the main role of digitizing the EMG signals [15], reducing the data flow towards the subsequent blocks, while preserving information useful for the MFCV estimation [17]. The resulting bit-streams (one per monitored channel) are sent the MFCV Control Unit, which estimates the conduction velocity. 

The MFCV Control Unit can be further divided into two internal blocks: the ϑ -Computing block and the Bluetooth Manager. The ϑ -Computing block embeds a single XNOR gate to compare the bit streams from the two EMGs, extracting the action potentials propagation time (ϑ) along the same muscle fiber. Then, it is provided to the Bluetooth Manager that computes the ratio between the inter-electrode distance and the time delay (ϑ) and manages the extracted MFCV for external transmission. 

A block for the footswitches signals analysis operates in parallel with the MFCV Control Unit, managing the overall system synchronization. It sets the time instant for the enabling of the MFCV computing or Bluetooth transmissions.

#### 2.2.1. Acquisition Interface

The Acquisition Interface is composed by four EMG electrodes, two for each leg, and two footswitches, one per foot. Each EMG electrodes pair is positioned on the subject’s lateral gastrocnemius, as seen in Figure 1. Here, the electrodes pair is labelled as the couple {1, 2} inside the white circles. The single footswitch (green plantar – Figure 1) consists of four Force-Sensitive Resistors (FSR), placed in specific positions under each foot.

Each FSR embeds a read-out circuit that provides in output a different voltage depending on the site under pressure, as shown in Table 1. Then, the footswitch sums the voltages provided by the FSR read-outs, returning a voltage that ranges between 0 V and 1.875 V. 

The sensing system works at sampling frequency respectively of 2 kHz for the EMG signals and 72 Hz for the footswitches, with a resolution of 16 bit. In addition, the EMG signals are numerically band-passed between 10–500 Hz to avoid artifacts [10].

#### 2.2.2. Sensors Placement

**EMG positioning.** The most critical point of MFCV estimation, via surface EMGs, lies in the difficulty of finding the optimal position of the electrodes for monitoring [10,14]. The optimal placement arrangement for MFCV estimation depends on knowledge of muscle anatomy and, in particular, the identification of the Tendinous (TZ) and Innervation Zones (IZ) [14]. 

**Figure 2:**
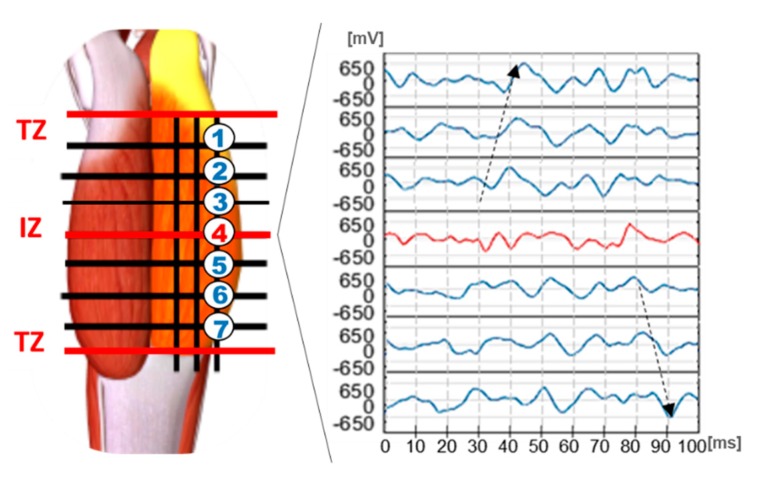
Surface Electromyography (EMG) positioning lattice with detailed EMG acquisition on seven selected sites. The waveforms refer to an experimental acquisition (Subset 2 – dataset: gait exercise- time window: 100 ms).

In this respect, Figure 2 shows a lateral Gastrocnemius, on which the prohibited areas (TZ and IZ) have been identified by three red solid lines; a black matrix defines the zones available for placement [14]. According to Figure 2, the EMG electrodes must be placed along the muscle fiber between the TZ and IZ. For this purpose, the guidelines in [14] provide an important support to identify these muscle areas.

The right side of Figure 2 shows the EMG signals acquired by the seven electrodes placed on the same column (Electrodes 1–7). Here, the action potentials propagation directions are indicated by black arrows. The EMGs between the TZ and IZ areas have coherent behaviors, i.e., they show the same peak that propagates along the muscle. 

**FSR positioning.** To provide a complete description of the foot position during a gait or during squat exercises, the four FSR-sensors, which compose the single wireless footswitch, are placed on four different under-foot points: heel, fifth metatarsus, first metatarsus, and big toe [18]. 

In the following, the FSR on the heel is labelled H, the FSR on the first and fifth metatarsus are defined M1 and M5, respectively. The fourth sensor on the big toe is named A. Each FSR works as a switch, where the state CLOSE or OPEN depends on the exceeding of a pressure threshold. 

All the sensors return 0V when in the OPEN state, while different voltages are linked to the CLOSE state of each sensor, as shown in Table 1. When more sensors are in the CLOSE state at the same time, the footswitch provides the sum of all the voltages as output. For instance, Table 2 summarizes the voltage values returned by the system during the proposed exercises.

##### User-Centered Surface Electromyography (EMG) Placement Calibration

The first step of the EMG placement algorithm consists of finding an optimal inter-electrode distance, which ensures the linearization of the muscle fiber segment and the physical implementation (i.e., electrodes gel ring). In this work, an inter-electrode distance D = 23 mm has been selected according to [14]. Then, two matrices between TZ and IZ have been traced considering a distance D between each contiguous elements, as seen in Figure 2. The placement algorithm takes into the account that the action potentials propagate along the muscle fiber by keeping the same waveform, but with an unavoidable amplitude reduction [10,11,12,13,14]. For this reason, an automatic placement system could exploit a covariance-based algorithm. In fact, the covariance permits to quantify the degree of similarity between two EMG signals in term of waveform and magnitude. Ideally, we expect that an optimal positioning returns a high covariance value, while the covariance is low when the placement includes the TZ and IZ. In these zones the action potential assumes different waveforms, demonstrated by the red waveforms in Figure 2. 

According to the covariance theory, the proposed system implements an algorithm that compares the EMG signals from two contiguous electrode sites of the same column, e.g., 1 and 2 seen in Figure 2. 

The algorithm starts considering the signals from the pair of electrodes: {1, 2}, as numbered in Figure 2. For sake of clarity, the signals acquired from these sites have been named EMG_1_ and EMG_2_, respectively. During the calibration phase, subjects are asked to stay on the toes to keep a static contraction of the Gastrocnemius for about 2 s. 

During this phase, the EMG samples acquired from each monitored channel (i.e., four sites) are used to define four threshold values (one per channel). To extract these thresholds (Thr), the system considers four vectors that contain squared EMG samples from the isotonic contraction. For each vector, the maximum is derived as a reference point, and 80% of this value is identified as the channel-related threshold. The 80% of the maximum represents the functional limit obtained by a statistical characterization of the typical amplitude reduction of the motor unit potentials propagation.

After the definition of the thresholds, the subject is asked to carry out another contraction. 

A single pair of electrodes is used as an example: {EMG1, EMG2}. Because of the propagation direction (from bottom to top as shown by the black arrow in Figure 2), only the bottom electrode (i.e., EMG2) is initially monitored by the system. When an EMG2 sample satisfies the conditions:

(2) $${\mathrm{EMG}}_{2}{}^{2}\;>\;\mathrm{Thr}$$

(3) $${\mathrm{EMG}}_{2}\;>\;0$$

The system starts an on-going procedure to find the maximum value of the signal by electrode 2 (EMG_2MAX_), assessing the change of waveform slope. 

Once the EMG_2MAX_ is found, the system acquires the EMG_1_ samples for a physiological time range (i.e., 4 ms [15]). 

The proposed system extracts on this time window the EMG_1MAX_ value. Once both EMG_1MAX_ and EMG_2MAX_ are defined, the relative error δ is extracted as:

(4) $$\delta =\left|\frac{{\mathrm{EMG}}_{2\mathrm{MAX}}-{\mathrm{EMG}}_{1\mathrm{MAX}}}{{\mathrm{EMG}}_{2\mathrm{MAX}}}\right|$$

A low δ value corresponds to a high degree of similarity between the signals (high covariance). If δ < 20% an optimal electrode positioning has been reached and the system provides the subject an audio/visual feedback (e.g., blinking LED or a buzzer sound). Otherwise, a different electrodes positioning is required (e.g., placing the electrodes on the sites pair {2, 3}).

#### 2.2.3. One-Bit Word Generator

The 1-bit word Generator associates the 16-bit EMG signals with muscle tone-inspired binary codes. Specifically, to realize these bit-streams, the EMG signal undergoes a digitization process based on a dynamic-threshold algorithm [19,20]. 

As a first step, each collected EMG signal is squared and stored in two circular registers. The first register stores M = 1024 samples at a sampling rate of 2 ksps (i.e., 500 ms of acquisition) that are used to compute the average signal power. The average signal power constitutes the global threshold (G-threshold in the following). The second register stores the last N = 8 samples at 2 ksps (i.e., 4 ms of acquisition) of the previous one. These samples are used to compute again the average signal power but on a shorter time window, defining a local average (L-power in the following). This process is updated for each sample. 

For the i-th sample, the binarized EMG (EMGb in the following) assumes the value “1” if L-power > G-threshold, otherwise “0”. The working principle of the dynamic thresholding algorithm is detailed in our previous works [19,20].

#### 2.2.4. Muscle Fiber Conduction Velocity (MFCV) Control Unit

The MFCV estimation from bit-streamed EMG signals is entrusted to the MFCV Control Unit.

In consideration of the propagation direction in the upper part of Gastrocnemius, as seen in Figure 2, the binary waveform associated to the bottom electrode of the chosen pair is named EMGb_A_; the bit stream linked to the top electrode is named EMGb_B_. The ϑ Computing Block stores in parallel both the EMGb signals in two dedicated shift-registers (REG_A_ and REG_B_).

These registers have N_b_ = 602 memory cells of 1 bit. Because the Analog-to-Digital Converter (ADC) sampling rate is 2 ksps, each register realizes an observation window of 301 ms. When the filling phase is over, the registers’ bits in the same position undergo iterative comparisons through a single XNOR gate. 

When the compared bits are equal to each other (${\mathrm{EMGb}}_{A}\left(i\right)={\mathrm{EMGb}}_{B}\left(i\right))$, the XNOR output is ‘1’, otherwise ‘0’. 

The number of times that the XNOR returns ‘1’, named in the following Number Equal Bit (**NEB**), indicates the degree of similarity between the EMGb_A_ and EMGb_B_:

(5) $$NEB\left(i\right)=\sum_{j=1}^{N_{b}}{\mathrm{EMGb}}_{A}\left(j\right)\bar{⊕}{\mathrm{EMGb}}_{B}\left(j\right)$$

with *i* = 1, …, N_b_ number of NEB values. After n*N_b_ comparisons, with n = 1, 2, …, N_b_, a ‘0’ is appended in the Least Significant Bit (LSB) of the shift register REG_B_ (e.g., element REG_B_[0]). This latter operation realizes a sequence shift of 1 bit, which corresponds to a temporal right shift of 0.5 ms. For each N_b_ comparison, the extracted NEB is compared with the previously extracted one to find the maximum NEB value. The number of comparison cycles that are needed to define the maximum NEB value defines the index that contains the maximum cross correlation level (i_NEB_MAX_).

The i_NEB_MAX_ value that can range between 1 ÷ N_b_ = 601 (0.5 ÷ 300.5 ms) and allows the ϑ value to be estimated through the equation:

(6) $$\vartheta =T_{s}•(i_{\mathrm{NEB}-\mathrm{MAX}}\;-1)$$

where $T_{s}=1/$f_s_ with f_s_ sampling frequency. Finally, the estimated ϑ value is provided to the Bluetooth Management Unit for external transmission.

#### 2.2.5. Bluetooth Management Unit

The time delay (ϑ) provided by the MFCV Control Unit to the Bluetooth Management Unit is used to calculate the MFCV value by the ratio between D (chosen inter-electrode distance) and ϑ estimated value. The result of the ratio, which corresponds to the MFCV in [m/s], is transmitted by the FPGA to an external device using a FSM-handled Universal Asynchronous Receiver-Transmitter (UART) protocol.

#### 2.2.6. Synchro Unit

The Synchro Unit triggers the activity of the ϑ Computing Block and the Bluetooth Manager Block by recognizing specific feet positions. For the MFCV estimation in a gait evaluation context, the Synchro Unit identifies two specific gait phases: one to start the ϑ estimation and another one to send the data outside the platform, taking care of the system computing times. As shown in Table 2, a gait phase can be uniquely defined by referring to the sum of four FSRs voltages. The system recognizes gait phases by using six different thresholds, one for each phase.

According to Table 2, each phase can be defined by one or more FSRs activation combinations. The recognition values are obtained considering the 90% of the minimum voltage value that identifies the monitored gait phase. As mentioned above, in this work, the investigated muscle for the MFCV measure is the Gastrocnemius. This muscle uniquely activates only in the Midstance phase, as shown in Table 3. A threshold of 1.575 V (90%@1.75 V) identifies the Midstance. During the gait, the Midstance enables the ϑ Computing Unit, while the Bluetooth Manager is enabled by the Swing phase.

**Figure 3:**
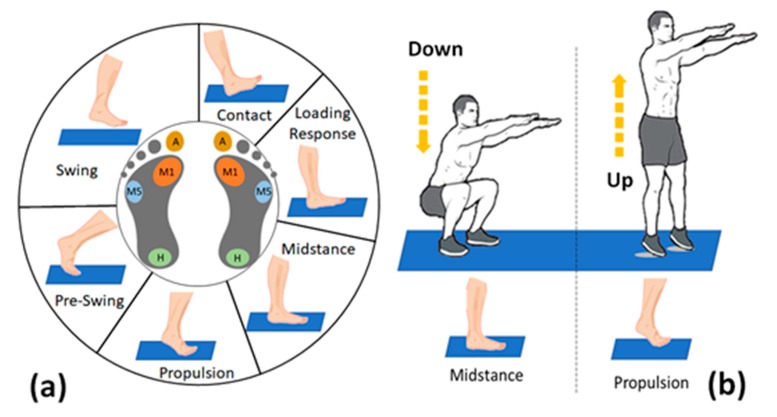
Footswitches involved during the exercises: (**a**) gait phase; (**b**) squat phase.

For the MFCV evaluation during the squat exercises, as shown in Figure 3b, the phases that enable the θ Computing Unit and the Bluetooth Management Unit are, respectively, the DOWN and the UP phases, as seen in Figure 3b.

During the DOWN (when the M1-M5-H sensors are in CLOSE mode), both the feet are in an equivalent gait Midstance. If the H sensor is not in CLOSE mode, the feet are considered in an equivalent gait Propulsion phase. In this latter case, the UP phase is detected and the Bluetooth Manager is enabled to transmit.

### 2.3. Field Programmable Gate Array (FPGA) Implementation Details

**Figure 4:**
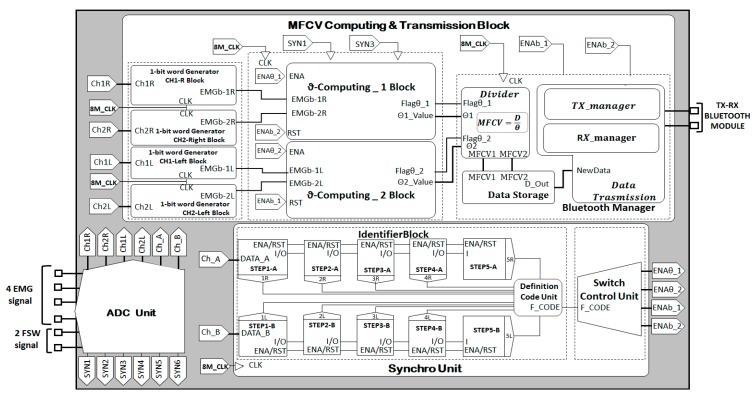
Block diagram of the muscle fiber conduction velocity (MFCV) estimator FPGA design.

The algorithm for the MFCV estimation, detailed in its working principle in the previous section, has been fully implemented on the Altera Cyclone V FPGA SE-5CSEMA5F31C6N, which has been already validated in real-time, bio-signals processing [20]. Figure 4 shows the functional blocks that compose the architecture FPGA implementation.

The system I/O interface is composed by six bio-signals in input and two output MFCV estimates of 32 bits. The inputs are:

- Four EMG (two surface electrodes per leg), sampling rate 2 kSa/s with a resolution of 16 bits.
- Two Footswitches signal (one per foot), sampling rate 2 kSa/s with a resolution of 16 bits. The footswitch bio signal can assume 11 possible values, as shown in the second column of Table 2.

According to the Figure 4, the first four signal in input (EMG signals) are divided into two groups, depending on the leg on which the electrodes are positioned: Ch1R and Ch2R refer to electrodes on right leg, while Ch1L and Ch2L refer to the left leg. Instead, the channels ChA and ChB refer to the signals provided by the left and right footswitch, respectively.

In addition, the system has a set of global signals:

- START/STOP are used to manage the functioning of the whole architecture;
- Reset handles the zeroing of all the registers and FSMs;
- 8M_clk is the chosen 8 MHz system clock. It is derived by the embedded 50 MHz FPGA clock (50M_clk). The 8M_clk is used to synchronize all internal activity;
- SYNi – i = 1…Nch, with NCh number of monitored EMG channels (four in our application) are the 2 kHz clocks that manage the inputs sampling rate. This clock is also derived by the 50M_clk.

#### 2.3.1. Identifier Block

As shown in Figure 4, this unit operates on the samples provided by the ChA and ChB lines (ChA for the right footswitch data and ChB left footswitch data). The dedicated clocks SYN5 and SYN6 defines the presence of a new data on these lines. In consideration of the working principle on a single channel, (i.e., ChA), when a new sample arrives, it is stored and compared with the data, which has been acquired during the previous clock cycle by the unit.

If the new sample is equal to the previous one, the system does not perform the computation (the gait phase is the same).

Otherwise, the data is compared with a cascade of five comparators (STEPi_A/B with i = 1...5 – Figure 4). An output Boolean flags can be extracted from each single comparator (i_A/B with i = 1...5) generating a thermometric code.

This code feeds the Definition Code Unit.

Starting from the rising edge of the SYN5 signal, each CLK_8M cycle, the Definition Code Unit provides in output a 10-bits sequence (5 bits for the right threshold system and 5 bits for the left one), which is named F_CODE. The Switch Control Unit, to properly synchronize the other subsystems, uses the F_CODE.

#### 2.3.2. Switch Control Unit

The synchronization is entrusted to the Switch Control Unit, which operates downstream the Identifier Block. According to the movement biomechanics, detailed in Section II.E, the Switch Control Unit selectively enables the θ-Computing Block and the Bluetooth Manager Unit. The signals used to synchronize the above-mentioned functional blocks are labelled in Figure 4 as ENAθ_1/2 and ENAb_1/2.

If, during a gait, the subject is in the Midstance phase, ENAθ_1/2 goes ‘1’ enabling the θ-Computing Block. In contrast, when the Swing phase occurs, ENAb_1/2 goes ‘1’ enabling the data transmission by the Bluetooth Manager Unit. In a squat exercise, the ENAθ_1/2 goes ‘1’ during the Midstance, while ENAb_1/2 goes ‘1’ during the Propulsion.

#### 2.3.3. One-Bit Word Generator Block

**Figure 5:**
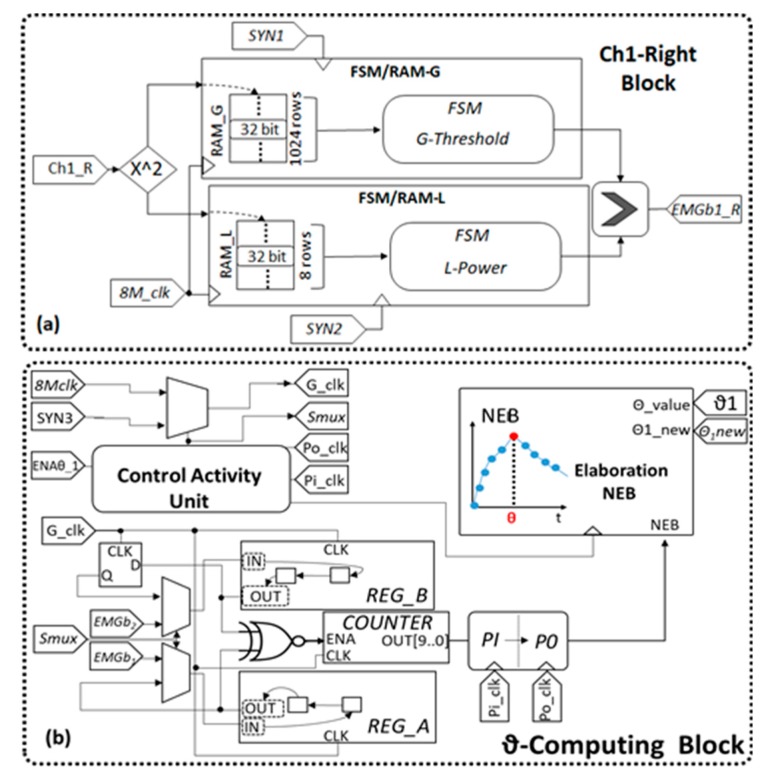
Functional block expansions: (**a**) Ch1-Right Block – (**b**) ϑ-Computing Block.

The 1-bit word Generator Block realizes a conversion of the EMG-acquired samples from 16 bits to 1 bit, realizing an EMG-based bitstream [19,20]. As show in Figure 5, each EMG channel has a dedicated conversion block. A total of four identical blocks operates in parallel.

Considering a single conversion block (e.g., the EMG1 on the right leg: Ch1-Right Block, as seen in Figure 5a), when a new EMG sample occurs, it is squared (32 bit) and parallelly provided to two FSM/RAM units. These units, named FSM/RAM-G and FSM/RAM-L, define, respectively, the average EMG power on a time window of 500 ms (dynamic threshold) and the EMG power on 4 ms (instantaneous power to be compared with the threshold).

The acquisitions windowing is realized by using dedicated RAMs. The RAM used to store the global power (RAM_G_) is constituted by 1024 words of 32 bits, while the instantaneous power RAM (RAM_L_) covers 4 ms with eight words of 32 bits. When a new sample is available to input, the data into the RAMs are shifted of one position and the new data is placed in the first memory cluster (First–In First-Out like functionality).

The two FSMs progressively sum the data stored in the dedicated RAMs. The sums are divided by eight to realize the L-Power, and by 1024 for the G-Threshold. The L-Power is compared with the G-Threshold. If the first one exceeds the dynamic threshold, the block generates a ‘1’, otherwise ‘0’. The resulting bitstream is named EMGbi with i=1…4 number of the monitored channel [20].

#### 2.3.4. θ-Computing Block

Downstream of the 1-bit word Generator Block, the system implements a unit that evaluates the degree of resemblance between two EMG*b*s related to the electrodes on the same leg. In particular, the EMG*b*s are analyzed in pairs, one for each leg: EMGb1-EMGb2_Right and EMGb1-EMGb2_Left. Considering, for instance, the branch that comprises the couple EMGb1-EMGb2_Right and the θ-Computing_1 Block, as shown in Figure 5b, when the Synchro Unit enables this block (ENAθ_1=‘1’and ENAb_1=‘0’), it evaluates the time delay (ϑ) between EMGb_1R_ and EMGb_2R_ sequences.

This operation can be broken down into two phases: acquisition and elaboration. During the first phase, the EMGb1 and EMGb2 bits are stored in the shift registers REG_A and REG_B. This phase is handled by the falling edges of a dedicated general signal clock G_Clk = SYN1 (2 kHz). Once the shift registers are full (after 602 cycles @2 kHz), the Control Activity Unit set to ‘1’ the bit flag Smux. It indicates that the acquisition phase is concluded and prepares the system for the elaboration phase. In this phase, the system clock passes from 2 kHz to 8 MHz (G_Clk = 8M_clk). The elaboration phase enables a pure feedback on the register REG_A, as seen in Figure 5b, and a delayed version of the stored vector for the register REG_B by using a D-Flip Flop (DFF), as seen in Figure 5b. During these operations, the bit sequence in REG_A remain unchanged, while the bit sequence in REG_B is right shifted. Then, as shown in Figure 5b, the shift registers LSBs are compared by an XNOR gate.

The resulting binary waveform powers up a 10 bits counter (ENA = ‘1’). It counts the number of G_clk clock cycle occurring during the ‘1’ state of the XNOR output. The extracted NEB value, for the i-th shift, is sent to the Elaboration NEB. This operation is cyclically repeated for 601 times, as stated in Equation (5). The clock commutation 2 kHz→8 MHz is handled by a glitch-free MUX (Control Activity Unit).

The Elaboration NEB unit is based on a dedicated FSM that iteratively compares the NEB value returns, updating a temporary memory with the maximum NEB value. It uses a clock signal that goes ‘1’ each 602 G_clk cycles. The i**_NEB-MAX_** (number of right shifts for REG_B) represents the wanted ϑ value as stated in Equation (6).

A pseudocode summarizes the described stages:

**INITIAL** i**_NEB-MAX_**← ‘0’,i←‘0’,j←‘0’; NEB**_MAX_**←‘0’,NEB ← ‘0’; **#Init Settings**

**WHILE** (i<602)

    i← i+1;

**WHILE** (j<602)              **# NEB VALUE DEFINITION**

**IF** (REG_A_[j]= REG_B_[j])      **# XNOR Comparison BIT-by-BIT**

          NEB ← NEB+1;     **# NEB accumulator**

          j←j+1;                **# Registers Shifting**


            **ELSE**
          

          j←j+1;                **# Registers Shifting**


            **END**
          


            **END**
          

**IF**(NEB[i]> NEB**_MAX_**)                   **# MAX NEB Comparison**

    i**_NEB-MAX_** ← i;                       **# i_neb_max_ Assignment**

    NEB**_MAX_** ← NEB;                   **# New MAX NEB Assignment**

    REG_A_← “0” & REG_A_[1÷601];            **# Shift Operation on REG b**

    j← ‘0’;                                 **# j index Initialization**

    NEB ← ‘0’;                          **# Temp var: NEB Initialization**


            **ELSE**
          

    REG_A_← “0” & REG_A_[1÷601];            **# Shift Operation on REG b**

    j← ‘0’;                             **# j index Initialization**

    NEB ← ‘0’;                          **# Temp var: NEB Initialization**


            **END**
          

  REG_B_← REG_B;_                            **# Reg A is Unaltered**


            **END**
          

**READ(**i**_NEB-MAX_**)                             **# Read the estimated time delay (θ)**

Finally, the extracted ϑ is provided to the Bluetooth Manager Unit.

#### 2.3.5. Bluetooth Manager Unit

Once the θ-Computing Block conclude all the internal operations, they set a Flagθ_1/2 to indicate that the ϑ values are available in output, and are ready to be sent out, as seen in Figure 4. The time delay from the right leg branch (θ1_Value) and the same from the left leg branch (θ2_Value)are passed to the Divider Unit. This unit realizes the ratio between D and ϑ_Values. The MFCV obtained values, MFCV1 and MFCV2, are stored in dedicated RAMs (Data Storage).

When the ENAb_1/2 goes ‘1’, the stored data are sent to the Data Transmission Unit. This unit handles the data, preparing them for the transmission to an external module (HC-05). The system uses for the data transceiver the Universal Asynchronous Receiver-Transmitter (UART) protocol [21]. Each transmitted data packet is made up of six frames of 10 bits. Of these 10 bits, 8 bits contain the data to be sent, one bit is placed before and one after the string [21]. Specifically, the Most Significant Bit (MSB) is preceded by a ‘0’, and the LSB is followed by a ‘1’.

In our application, the first and the sixth frames contain the number (254)_10_, with the aim of starting and stopping the communication; the frames in the middle, from the second to the fifth, contain the MFCV value. The FPGA has been programmed to operate with a baud rate of 9600 baud.

## 3. Results

This section is dedicated to the implementation and testing of the proposed FPGA-based MFCV estimator in the context of walking assessment and fatigue monitoring.

The system was tested on six healthy volunteers (aged 24 ± 3) all students of Politecnico di Bari, Italy. Before starting the experimental sessions, all the participants signed the informed consent. Research procedures were in accordance with the Declaration of Helsinki and was approved by the Local Ethical Committee (Protocol n. 2019_0025904).

All the subjects were involved in 10-m slow and power walk protocols [3] for the dynamic MFCV estimation during the gait. Instead, two subjects from the original dataset (both 26 aged) carried out squat exercises to allow the MFCV estimation in a fatigue monitoring field.

The Section 3.1 and Section 3.2 validate our FPGA based platform in some clinical or ordinary life applications (e.g., dynamic contractions in walking or in squat exercises), comparing the system results with the field literature [5].

Finally, the Section 3.3 and Section 3.4 focus their attention on the FPGA performances, in a future perspective of ASIC implementation: resources utilization, timing and power consumption.

### 3.1. In Vivo MFCV Measures: Gait

**Figure 6:**
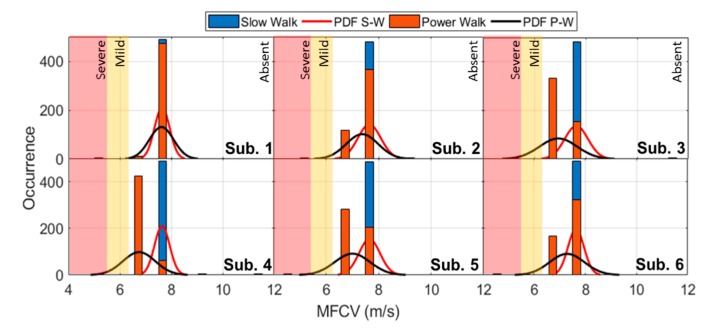
Subject-by-subject MFCV values occurrence and probability density functions in 10-m slow walk protocol (blue histogram) and considering a power walk (red histogram).

The subjects were asked to walk in a roundtrip path of 10 m for 500 steps. Each step, in its midstance phase, was used as a trigger for the MFCV estimation, realizing a dataset of 3000 values. In Figure 6, the blue histograms show the occurrence of the MFCV values for each subject; the red indicates the probability density function. As expected for healthy subjects, the overall evaluation of the system is, on average, MFCV = 7.6 m/s, with a standard deviation of only ±0.36 m/s. In the same operative conditions and for healthy subjects, the literature study [5] provides on the Gastrocnemius of 45 patients, a mean value of MFCV = 7.51 m/s (standard deviation of ±0.31 m/s). According to [3], it is possible to define two MFCV bands for the detection of early (mild: yellow) and late-stage (severe: red) diabetic neuropathy. The bands are shown in Figure 6, highlighting the clinical coherence of the estimates. Furthermore, the system was tested in a modified 10-m protocol, where the subjects were asked to perform the “power walk” for 500 steps, halving the test time. The 500 steps were divided in blocks of 100 steps, interspersed with a brief pause (~120 s).

**Figure 7:**
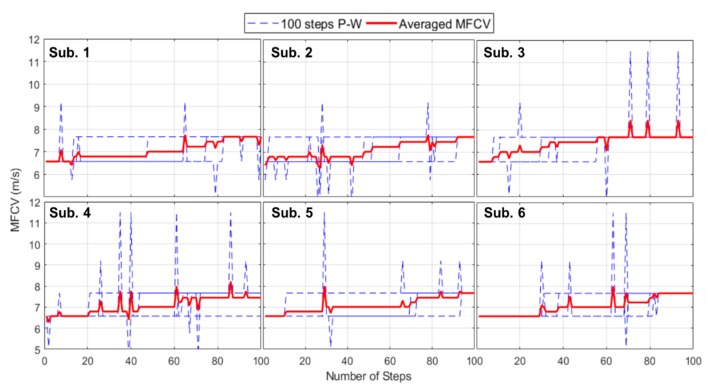
Subject-by-subject MFCV behavior in a power walk comprising five blocks of 100 steps (dashed blue line). In red, the average of the MFCV.

In this second task, shown as red histograms in Figure 6 (with the probability density function, PDF, in black), the occurrence of the MFCV is scattered on two contiguous values, reaching an MFCV = 7.16 m/s (standard deviation: ±0.63 m/s). The value is still clinically coherent with the literature [22], showing in time domain an increasing behavior. As shown in Figure 7 in “power walk”, the MFCV value increases from 6.57 m/s to 7.67 m/s during 100 steps. After the pause, the MFCV returns to 6.57 m/s.

**Figure 8:**
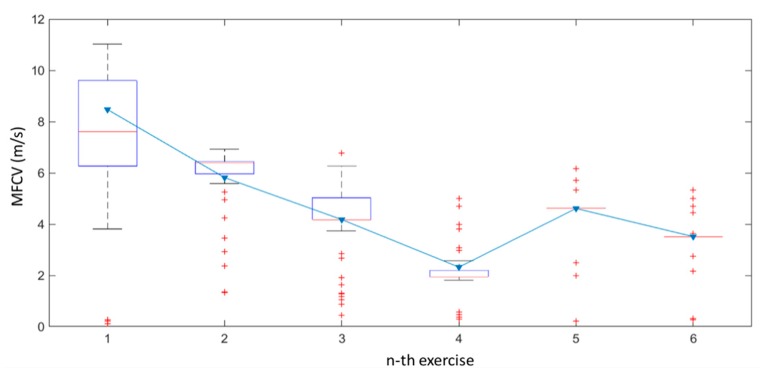
MFCV values behavior during squat exercise.

The MFCV averages are shown in Figure 8 with red lines indicating five sessions and six subjects. The behavior reflects a typical phenomenon known as acidosis [20].

### 3.2. In Vivo MFCV Measures: Fatigue

Two subjects from the general dataset participated in the MFCV assessment for the fatigue monitoring by carrying out a series of squat exercises without additional weights. The squat modality, as shown in Figure 3b, requires 30 squats for each run.

In overall, six runs were realized for each subject, interspersed by a relaxation pause of 10 s. The MFCV estimations in a fatigue monitoring context are shown in Figure 7.

The statistical analysis of the data provided during each single consecutive run has been represented in a boxplot. On the basis of data from both subjects, Figure 8 shows that the extracted MFCV values are sparser, with a median value of 7.67 m/s when the subject is rested, while are more coherent around the value 4.6 m/s (neglecting the outliers) when the subject is overdriven. Figure 8 also contains a solid blue line, showing the decreasing behavior of the MFCV values average when the fatigue phenomenon occurs. This decrement matches the theory in literature [8,9]. Typically, the average decreases from 8.51 m/s to 4.60 m/s (outliers comprised in the computing).

### 3.3. FPGA Resources Utilization and Timing Requirements

**Figure 9:**
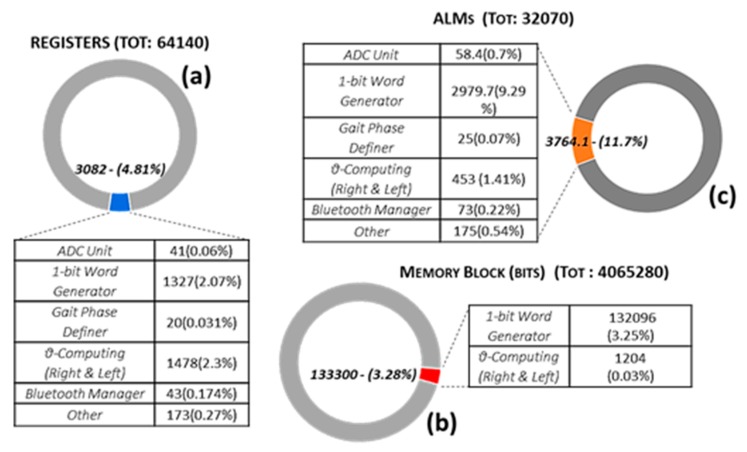
FPGA resources utilization: (**a**) Registers - (**b**) Memory Block - (**c**) Adaptive Logic Modules. The Other label indicates the surrounding circuitry.

During the acquisition context, the system involves approximately 32 kbps on each ADC channel ($2000\frac{Sa}{s}\cdot 16\frac{bit}{Sa}$), overall 192 kbps (six channels). The output data, for a transmission continuative of 1 s, are 9.6 kbit. The tables in Figure 9 report the resource used by the system for each unit.

Overall, the system uses 11.73% of ALMs available (3764.1/32070), 3.36% of memory blocks (133300/4065280), as well as 4.81% of available registers (3082/64140) and <4.5% of available interconnections (wires). Here, the label *Other* defines the surrounding circuitry.

Starting from the instant in which the Midstance phase is recognized, 301 ms are necessary to complete the insertion of the bits in the θ-Computing Block registers, and 63.5 ± 0.25 ms to define the ϑ value.

Overall, 364.5 ± 0.25 ms are necessary. The number of clock cycles necessary to complete the registers filling operations are 602 cycles@2 kHz, while, in the worst case, 362,403 cycles at 8MHz and one at 2 kHz (602 iterative comparisons*602 temporal shifts). Using the time window of 1 s from Midstance recognition, on a single step, the 1-bit Word Generator operates for 100% of the time, and the operative block, and θ-Computing Unit work for the 34.68% of the time. Others, 34 cycles at 8 MHz, are required to realize the ratio between space and latency to generate the MFCV value (Divider). The Data Transmission Unit requires 57 cycles at 8 MHz (about 7.13 µs) to realize the data pack and 5.93 ms at 9600 baud for the frames transmission. Others, such as 15 ms, are added by the wireless recording system.

### 3.4. Power Consumption

As previously stated, the proposed system is a wearable, oriented digital architecture. In this respect, for power consumption estimation, the system must be divided into two subsystems: the acquisition interface (EMG and Footswitches) and the elaboration platform (FPGA).

In consideration of the former system, the wireless EMG sensors and footswitches operate with a power supply voltage of 4 V (lithium battery of 28 × 20 × 12 mm), absorbing 40 mW with a sampling rate of 2 ksps. The devices also have an apparent radiated power (ARP) of 0.45 mW at 2.4 MHz.

In a continuous-mode operation, the EMG/footswitches nodes are able to send data for about 12 h.

Because the computation unit is fully embedded on the Altera Cyclone V FPGA SE-5CSEMA5F31C6N FPGA, the power consumptions estimate have been provided by the PowerPlay Power Analyzer tool, by keeping a “high” power estimation confidence [23].

The low resources utilization, jointly with the light I/O data flow, lead to static as well as a dynamic powers estimation lower than 0.1mW, in an ASIC implementation framework [24].

However, the use of a prototyping board with TTL interfaces (DE1-SoC Terasic board) led to a static power consumption of P_STATIC_ = 453.24 mW with V_CC_ = 12 V at 25 °C (no heat sink with still air).

According to the PowerPlay Power Analyzer [23] notation, the P_STATIC_ comprises the thermal power dissipated on chip, independent of user clocks. It includes the leakage power from all FPGA functional blocks, except for I/O dc bias [23]. Table 4 details every contribution to the P_STATIC._ Referring the following data to V_Core_ = 1.1 V, the system dynamically dissipates about P_DYN_ = 28.60 mW working on 603 cycles at 2kHz and 362,494 cycles at 8MHz. Details regarding the entities that contribute to the P_DYN_ is reported in Table 4. Finally, the computation comprises the PLL unit, which consumes 11.55 mW with a dedicated V_CCPLL_ = 2.5 V. The power dissipated dynamically were obtained considering a test bench of 1s.

## 4. Conclusions

The paper outlines the design and implementation of an FPGA-based platform for the MFCV estimation during ordinary movements (i.e., gait) and fatigue monitoring. The system is based on a fast cross-correlation procedure based on a single XNOR gate. The MFCV computing algorithm shows a very light hardware resource utilization, with only the 11.73% of the ALMs available on the Altera Cyclone V FPGA, 3.36% of memory blocks, and 4.81% of registers available, without need for processor interactions.

The proposed system also implements an easy electrodes positioning tool, which allows the patient to place EMG probes without medical support. Table 5 details a comparison among the proposed work with highly used solutions proposed by the state of the field.

The platform has been validated on six subjects (aged 24 ± 3) involved in a 10-m walk protocol and squat exercise. In consideration of the MFCV estimations in a walking evaluation context, the proposed platform returns an MFCV = 7.6 m/s ± 0.36 m/s, matching clinical literature (MFCV = 7.51 m/s ± 0.31 m/s).

Furthermore, the MFCV values extracted during the fatigue monitoring show an expected descending trend of the conduction velocity when fatigue occurs.

The system manifests a wide range of applicability, allowing the estimation of the MFCV during different types of exercises, leaving the structure unaltered.

In practical applications, the proposed system is able to provide outputs (i.e., MFCV data transmitted via Bluetooth) in about 370 ms, ensuring up to 12 h of operation in a continuous mode. In this context, the system showed a dynamic power dissipation of about 69.05 mW, of which 28.60 mW are dissipated from the elaboration platform (FPGA), while the remaining 40.45 mW are from the acquisition devices (EMG/footswitch).

## Figures and Tables

**Table 1:** Voltages provided by the Force-Sensitive Resistors (FSR) sensors in close mode.

FSR Sensor	Position	Voltage in Close Mode (V)
H	Heel	1.00
M5	Fifth Metatarsus	0.50
M1	First Metatarsus	0.25
A	Big Toe	0.125

**Table 2:** Voltages provided by the FSR sensors in close mode.

Gait Phase	Footswitches Voltage (V)	Threshold (V)
P6 – Midstance	M1 + M5 + H → 1.75	1.575
	A + M1 + M5 + H → 1.875	
P5- Loading Response	A + M5 + H → 1.375	1.125
	M5 + H → 1.25	
	M1 + H → 1.5	
P4- Contact	H → 1	0.9
P3- Propulsion	M1 + M5→ 0.75	0.675
	A + M1 + M5→ 0.875	
P2- Pre-Swing	A + M1 → 0.625	0.113
	A + M5 → 0.375	
P1- Swing	All sensors in OPEN mode	0

**Table 3:** Muscles involved by the specific gait phase.

Gait Phase	Threshold (V)
P6 – Midstance	**Gastrocnemius**, Soleus
P5- Loading Response	Adductor, Peroneal, Tibialis rear
P4- Contact	Quadriceps, Tibialis ant., Gluteus
P3- Propulsion	Tibial rear, Peroneal, Finger flexors
P2- Pre-Swing	Adductor, Femoral Rectus
P1- Swing	Tibialis, Quadriceps

**Table 4:** Power Consumption Features.

**Static Power**	**Power Dissipation (mW)**	**Operative Conditions**
Thermal Power	416.6	V_CCaux_ = 2.5 V
Leakage Power	10.5	V_Core_ = 1.1 V
I/O Management	25.5	V_CCaux_ = 2.5 V
**Dynamic Power**	**Power Dissipation (mW)**	**Operative Conditions**
ADC	1.25	V_CCaux_= 2.5 V - 2000 cycles @2 kHz
PLL Unit	11.55	V_CCPLL_ = 2.5 V
I/O dynamic Management	3.6	V_Core_ = 1.1 V 603 cycles@2 kHz and 362494 cycles@8 MHz
Register cells	1.04	
Combinational blocks	0.04	
Memory 10kb	11.12	

**Table 5:** State of the field: Comparison Table.

Parameters\Work		This Work	[8]	[11]	[12]
Platform		EMG Footswitch FPGA	EMG ASIC	EMG μC	EMG Force Controlled Chair PC
Applicability	OL ^1^	✔	✔	✘	✘
	Clin ^2^	✔	✔	✔	✔
EMG Stimulation		Voluntary	Voluntary	Electrical	Voluntary
Involved Limb		Leg	Arm	Arm	Arm
Num. of electrodes		4	2	8	64 (array)
Positioning Assistance		✔	✘	✘	✘
Computing Method		Single XNOR cross-correlation	On-going cross-correlation	Cross-correlation	Delay-locked loop (DLL)
Timing ^3^		Real-time	Real-time	On-line computed	On-line computed
ϑ Resolution (ms@f_clk_) ^4^		0.5 ms 2 kHz	0.5 ms @2 kHz	50 ms @1 kHz	1 ms @1 kHz
Application		Fatigue OL MFCV Monitoring	Fatigue	MFCV Monitoring	MFCV Monitoring

^1^ OL: Ordinary Life applicability. ^2^ Clinical applicability. ^3^ Real-time requests for acquisition+computation time <400 ms. ^4^ The ϑ resolution is upper limited by 2 kHz (sampling frequency of the presented work).
